# Digital confidence mediates the impact of visual impairment on daily internet use in older adults

**DOI:** 10.1093/geroni/igaf122.4304

**Published:** 2025-12-31

**Authors:** Haruno Suzuki, Thomas Hoffmann, Heather Leutwyler, Margaret Wallhagen

**Affiliations:** University of California, San Francisco, San Francisco, California, United States; University of California, San Francisco, San Francisco, California, United States; University of California, San Francisco, San Francisco, California, United States; University of California, San Francisco, San Francisco, California, United States

## Abstract

Older adults with visual impairment (VI) often experience barriers to engaging with digital technologies, yet remain underrepresented in digital health research. Confidence in using technology may mediate the association between VI and digital behaviors. This study aimed to 1) explore factors associated with digital behaviors among older adults with VI and 2) examine whether confidence in using technology mediates the relationship between VI and digital behaviors in older adults. The study used cross-sectional, nationally representative data from HINTS 2024. The sample included respondents aged 60 and older (N = 3,149). VI was defined using a self-reported item on visual difficulty. Outcomes included daily internet, smartphone, computer, and wearable device use in the past 12 months. Confidence in using applications/programs (e.g., Zoom) without help was the mediator. Multivariable logistic regression and mediation analyses with 1,000 bootstrap replications were adjusted for age, sex, race/ethnicity, education, marital status, self-rated health, and mobility impairment. Among older adults, 7.1% (N = 223) reported VI. Older age and lower educational attainment were significantly associated with lower odds of daily internet and smartphone use (P < 0.05). Statistically significant indirect (IE), direct (DE), and total effects (TEs) were observed for daily internet use (IE: OR = 0.92, 95% CI: 0.86–0.99, P = 0.017; DE: OR = 0.70, 95% CI: 0.53–0.93, P = 0.012; TE: OR = 0.65, 95% CI: 0.49–0.87, P = 0.003; proportion mediated: 20.3%, 95% CI: 1.9–38.7, P = 0.031), suggesting that VI reduces internet use partially through reduced confidence. Interventions targeting digital confidence, including personalized coaching, accessible interface, and peer support, may help bridge the digital divide in this population.

